# Metabolomic Profiling Reveals Mitochondrial-Derived Lipid Biomarkers That Drive Obesity-Associated Inflammation

**DOI:** 10.1371/journal.pone.0038812

**Published:** 2012-06-12

**Authors:** Brante P. Sampey, Alex J. Freemerman, Jimmy Zhang, Pei-Fen Kuan, Joseph A. Galanko, Thomas M. O'Connell, Olga R. Ilkayeva, Michael J. Muehlbauer, Robert D. Stevens, Christopher B. Newgard, Heather A. Brauer, Melissa A. Troester, Liza Makowski

**Affiliations:** 1 Department of Nutrition, Gillings School of Global Public Health, School of Medicine, University of North Carolina at Chapel Hill, Chapel Hill, North Carolina, United States of America; 2 Department of Biostatistics, Gillings School of Global Public Health, School of Medicine, University of North Carolina at Chapel Hill, Chapel Hill, North Carolina, United States of America; 3 Department of Epidemiology, Gillings School of Global Public Health, School of Medicine, University of North Carolina at Chapel Hill, Chapel Hill, North Carolina, United States of America; 4 Department of Medicine, University of North Carolina at Chapel Hill, Chapel Hill, North Carolina, United States of America; 5 Lineberger Comprehensive Cancer Center, University of North Carolina at Chapel Hill, Chapel Hill, North Carolina, United States of America; 6 Sarah W. Stedman Nutrition and Metabolism Center, Duke University Medical Center, Durham, North Carolina, United States of America; 7 Department of Pharmacology and Cancer Biology, Duke University Medical Center, Durham, North Carolina, United States of America; 8 LipoScience Inc., Raleigh, North Carolina, United States of America; State University of Rio de Janeiro, Biomedical Center, Institute of Biology, Brazil

## Abstract

Obesity has reached epidemic proportions worldwide. Several animal models of obesity exist, but studies are lacking that compare traditional lard-based high fat diets (HFD) to “Cafeteria diets" (CAF) consisting of nutrient poor human junk food. Our previous work demonstrated the rapid and severe obesogenic and inflammatory consequences of CAF compared to HFD including rapid weight gain, markers of Metabolic Syndrome, multi-tissue lipid accumulation, and dramatic inflammation. To identify potential mediators of CAF-induced obesity and Metabolic Syndrome, we used metabolomic analysis to profile serum, muscle, and white adipose from rats fed CAF, HFD, or standard control diets. Principle component analysis identified elevations in clusters of fatty acids and acylcarnitines. These increases in metabolites were associated with systemic mitochondrial dysfunction that paralleled weight gain, physiologic measures of Metabolic Syndrome, and tissue inflammation in CAF-fed rats. Spearman pairwise correlations between metabolites, physiologic, and histologic findings revealed strong correlations between elevated markers of inflammation in CAF-fed animals, measured as crown like structures in adipose, and specifically the pro-inflammatory saturated fatty acids and oxidation intermediates laurate and lauroyl carnitine. Treatment of bone marrow-derived macrophages with lauroyl carnitine polarized macrophages towards the M1 pro-inflammatory phenotype through downregulation of AMPK and secretion of pro-inflammatory cytokines. Results presented herein demonstrate that compared to a traditional HFD model, the CAF diet provides a robust model for diet-induced human obesity, which models Metabolic Syndrome-related mitochondrial dysfunction in serum, muscle, and adipose, along with pro-inflammatory metabolite alterations. These data also suggest that modifying the availability or metabolism of saturated fatty acids may limit the inflammation associated with obesity leading to Metabolic Syndrome.

## Introduction

Over 1 billion people worldwide and two-thirds of the US population are overweight or obese [1], [2]. Obesity and insulin resistance are strongly associated with the infiltration of adipose tissue by inflammatory cells [3]–[7]. The factors that induce immune cells to infiltrate adipose tissue are unknown, but may be related to free fatty acid release from adipocytes [8]. Lipolysis and serum non-esterified fatty acids (NEFA) are elevated with obesity, insulin resistance, trauma, or infection [9]–[12]. Furthermore, cytokines associated with obesity and insulin resistance such as tumor necrosis factor α (TNFα) can drive lipolysis and fatty acid release from adipose [13], [14].

HFD and saturated fatty acid intake correlate with Metabolic Syndrome [15]–[18]; while polyunsaturated fatty acids have been shown to improve insulin sensitivity, as well as lessen inflammation [19]–[22]. Saturated fatty acids are known to be pro-inflammatory through activating pattern recognition receptors including Toll-like receptors (TLR) and/or G-protein coupled receptors (GPCR) [23]. Therefore, we hypothesized that saturated fatty acids and metabolites derived from mitochondrial oxidation may be biomarkers that predict inflammatory response and insulin resistance in diet-induced obesity. Previous metabolomic work by our group identified biochemical markers or predictors of pathologic states such as Metabolic Syndrome, cardiovascular disease (CVD), insulin resistance, and other metabolic defects [24]–[28]. Here we have applied comprehensive metabolic profiling to compare a HFD that is typically used in diet-induced obesity studies with CAF diet, revealing diet-specific alterations in several metabolites, notably lauroyl carnitine. We then evaluated the effects of lauroyl carnitine on macrophage pro-inflammatory responses, with findings that implicate lauroyl carnitine as a mediator of obesity-induced inflammation.

**Figure 1:**
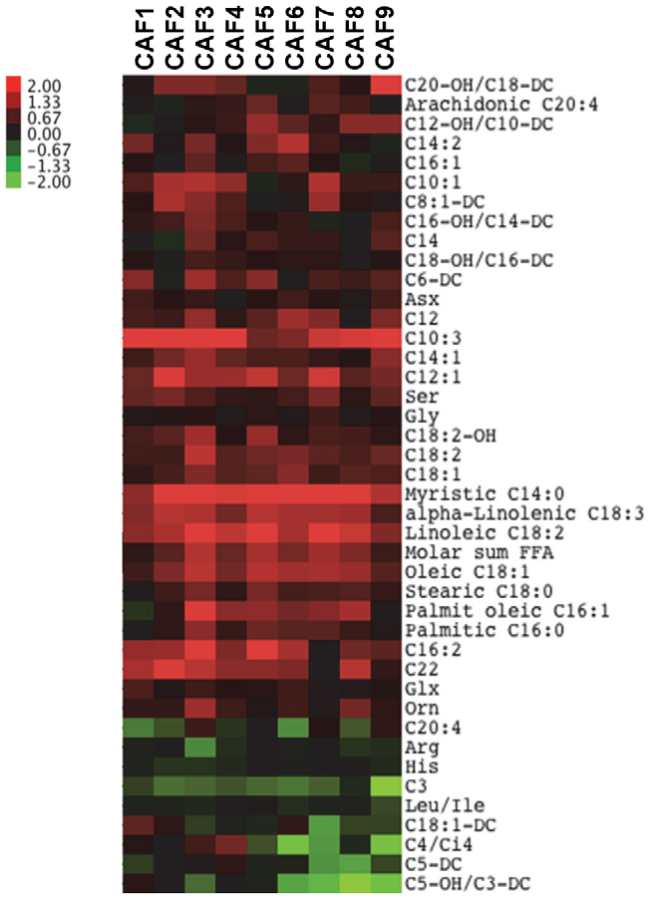
Serum accumulation of NEFA and acylcarnitine metabolites in CAF compared to SC-fed. Aged-matched male rats were fed diets for 10 weeks and serum was isolated in 6 hour-fasted rats. (n = 8 SC, 9 CAF). Amino acids and acylcarnitine mitochondrial intermediates were measured by LC-MS/MS. Non-esterified fatty acids (NEFA) were measured enzymatically. Metabolites indicate fold change of metabolites from CAF-fed serum compared to mean of SC-fed serum (CAF/SC) for each metabolite, which indicate accumulation (red) of many NEFAs and acylcarnitine species with decreases (green) in amino acids, arachidonoyl carnitine (C20∶4), and short chain acylcarnitines in the serum from CAF-fed rats (FDR 3.88%). See Table S4 for full names of metabolites.

## Materials and Methods

### Animals

This study was carried out in strict accordance with the recommendations in the Guide for the Care and Use of Laboratory Animals of the National Institutes of Health. The protocol was approved by the Committee on the Ethics of Animal Experiments of Duke University. Male Wistar rats (approximately 200 grams (g), 7–8 weeks old) (Harlan Laboratories, Dublin, VA) were housed 2 rats per cage in a 12 hour light/dark cycle and acclimated to the Duke animal housing facility on *ad libitum* undefined standard chow 7001 (“SC", Harlan Teklad Lab Animal Diets SC7001) for 2 weeks before assignment to one of four experimental diet groups. Upon initiation of experimental diets, rats (avg. 300 g, 9–10 weeks old) were either maintained on *ad libitum* SC as controls or switched to experimental diets: *ad libitum* defined 45% fat chow (High fat diet “HFD", Research Diets D06011802), the matched low fat chow control (Low fat diet “LFD" Research Diets D07010502), or a cafeteria diet (“CAF") with 3 human snack foods varied daily in addition to *ad libitum* SC as previously described [12]. Fat intake was the largest macronutrient alteration in CAF-fed rats, however simple carbohydrate consumption was also elevated over HFD and SC-fed rats groups [12]. Complete characterization of CAF, HFD, LFD and SC-fed rats including all diet details, food intake, weight gain, serum measures (insulin, glucose, total NEFA), other physiologic measures, adipose mass, as well as histology for liver, pancreas, epididymal white adipose tissue (eWAT), and brown adipose is presented in Sampey et al. [12]. Briefly summarizing that work, CAF-fed rats displayed the most severe obesity, glucose intolerance and inflammation compared to HFD, LFD and SC- fed rodents.

**Figure 2:**
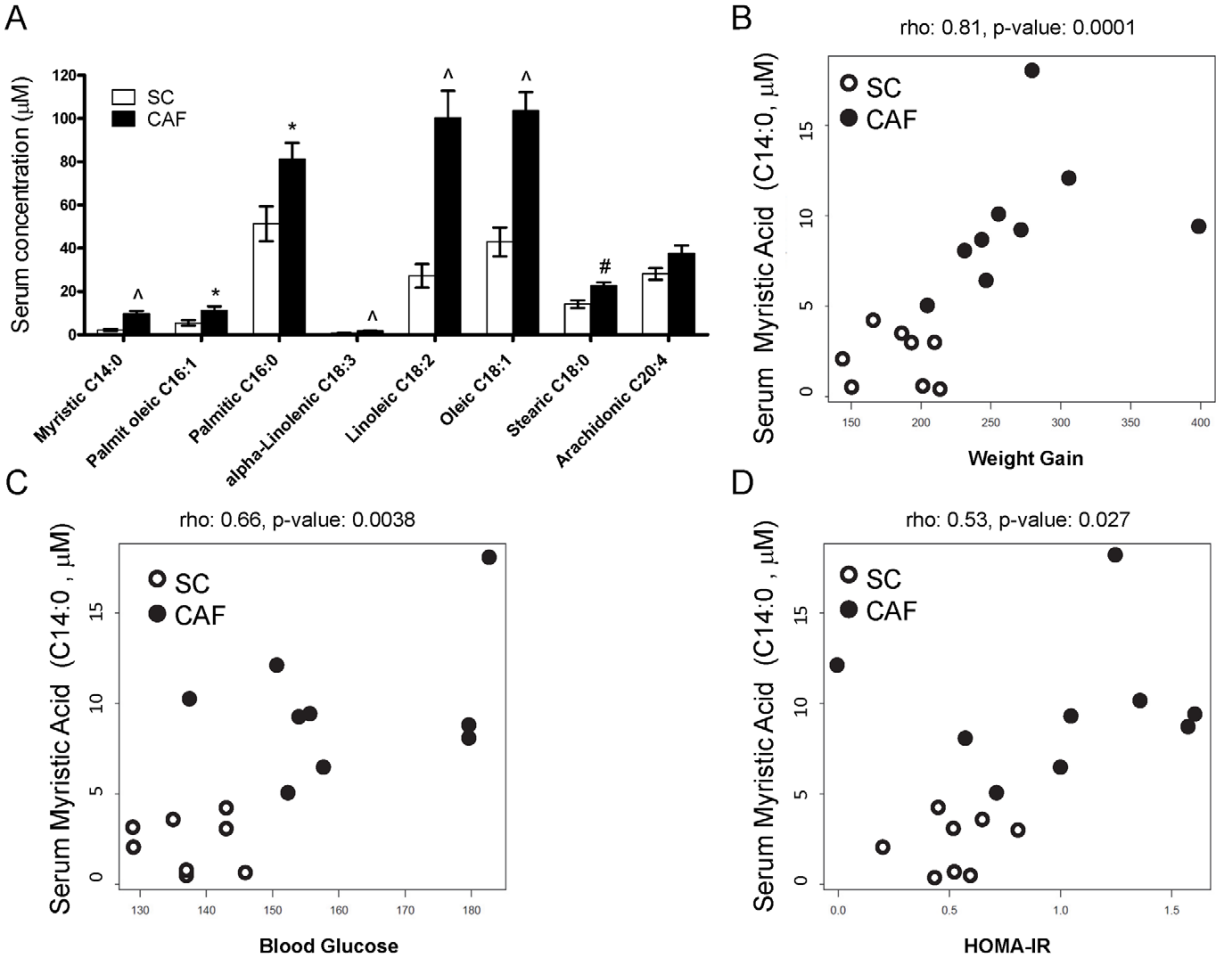
Serum myristate correlates to Metabolic Syndrome measures: weight gain, HOMA-IR, and blood glucose. Aged-matched male rats were fed diets for 10 weeks and serum was isolated in 6 hour-fasted rats and metabolites measured as in Figure 1 (n = 8 SC, 9 CAF). A) Serum concentrations of individual NEFAs indicate that of the eight fatty acids measured, seven were significantly elevated in CAF-fed rats versus SC controls. (*p = 0.04, #p = 0.01, ∧p<0.0001). B–D) Serum saturated fatty acid myristic acid (C14∶0) significantly correlated with weight gain (B), HOMA-IR (C) and blood glucose at sacrifice (D).

### Diet Studies

SC and CAF-fed rats were fed diets for 10 weeks and at sacrifice plasma, serum, or tissue was isolated for metabolomic analysis (study 1; n = 4–12 per diet, per group as indicated in Figures and Tables). Analyses included total NEFA, individual serum NEFA, acylcarnitine and amino acid metabolite profiling of serum, as well as acylcarnitine, organic acid, and amino acid profiling of liver and muscle. A subsequent group of experimental animals (n = 4–5 per diet) were fed SC, LFD, HFD, and CAF diets for 15 weeks and metabolites (amino acids, organic acids, acylcarnitines, and free carnitine) were isolated from eWAT (study 2).

**Figure 3:**
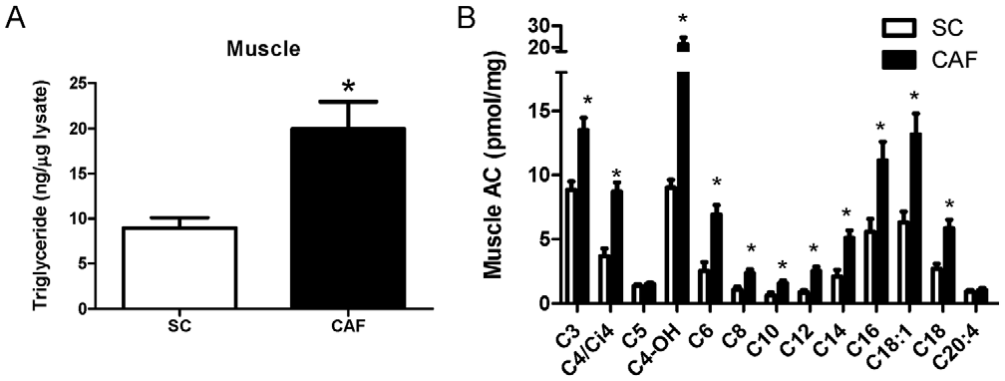
Lipotoxicity and mitochondrial dysfunction induced by CAF diet in muscle. Aged-matched male rats were fed diets for 15 weeks and tissue was isolated from animals sacrificed after a 6 hour fast. A) Muscle triglyceride levels were doubled in CAF-fed rats compared to SC-fed controls (*p<0.02, n = 4 SC, 5 CAF). B) Acylcarnitines accumulate in muscle of CAF-fed rats compared to SC controls (*p≤0.02). Metabolites measured and tissue isolation as in Figure 1. (n = 8 SC, 9 CAF for B). See Table S4 for full names of metabolites.

### Metabolomic Analysis

Serum (50 µL) and tissue [liver (100 mg), muscle (100 mg), or adipose (200 mg)] were used for metabolic profiling. Tissues were reconstituted in 4 volumes of deionized/distilled water for a 5-fold dilution. Samples were then immediately homogenized on ice at 25,000 RPM for 20 seconds (setting 5.5 on the VDI-12 homogenizer). Samples were mixed well by vortex and 100 μL was taken immediately for acylcarnitine (AC), amino acid (AA) and free carnitine analysis. Metabolomic measure of AC, AA, and carnitine is well-established in human and rodent models [27]–[35]. Methods for sample preparation are as described previously [27], [35]. Briefly, measurement of free carnitine, AC, and AA in serum and eWAT was completed by direct-injection electrospray tandem mass spectrometry (MS/MS), using a Micromass Quattro Micro liquid chromatography (LC)-MS system (Waters-Micromass, Milford, MA, USA) equipped with a model HTS-PAL 2777 auto sampler (Leap Technologies, Carrboro, NC, USA), a model 1525 HPLC solvent delivery system (Agilent Technologies, Palo Alto, CA, USA), and a data system running MassLynx 4.0 software (Waters Corporation, Milford, MA) as described [26], [27], [36]. NEFA were measured in serum enzymatically (Wako, Richmond, VA, USA) using the Hitachi 911 clinical chemistry analyzer (Hitachi, Tokyo, Japan) [12]. Table S4 provides the full names of acylcarnitines, organic acid, and amino acids analyzed.

**Figure 4:**
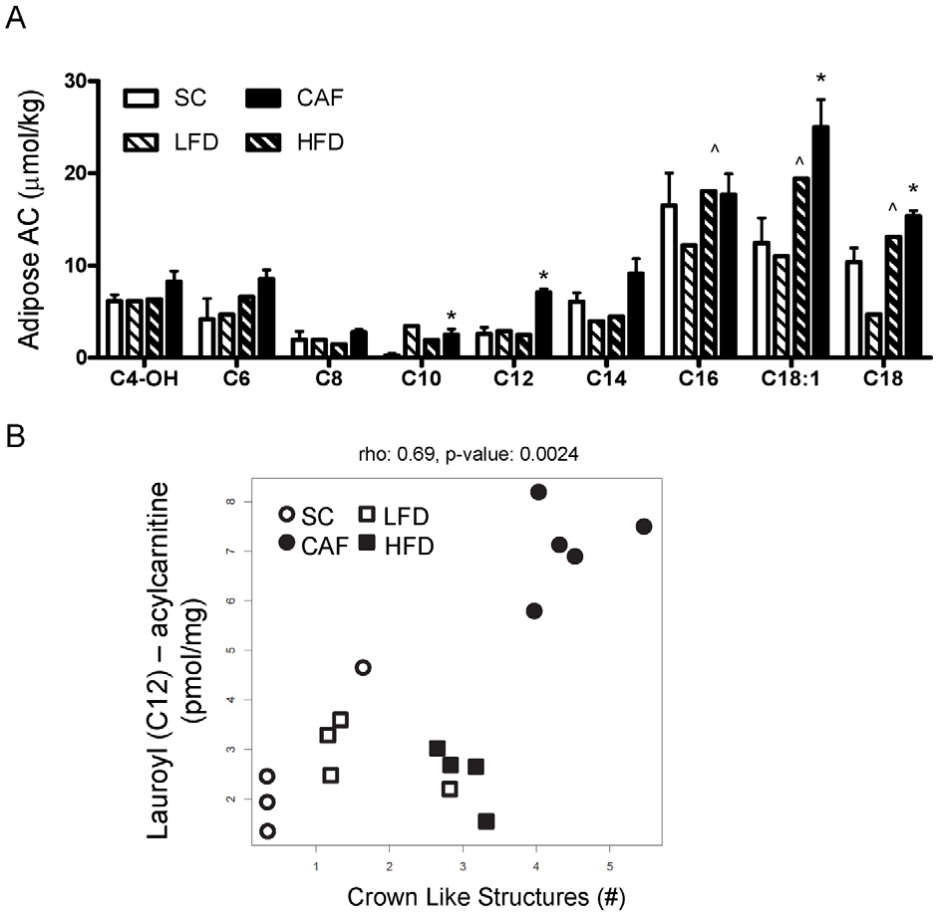
Severe mitochondrial dysfunction in CAF-fed white adipose tissue. A) Diet-induced mitochondrial dysfunction was evident in CAF-fed rat epididymal adipose, which was greater than dysfunction in HFD-fed animals. CAF-diet increased levels of multiple acylcarnitines when compared to HFD-, LFD-, and SC- fed animals demonstrating mitochondrial dysfunction (*p≤0.03 SC v. CAF; ∧p<0.02 LFD v HFD). B) Lauroyl carnitine (“LC", C12-AC) specifically elevated by CAF diet (A) is significantly correlated to crown like structure (CLS) accumulation, a hallmark of adipose inflammation. Aged-matched male rats were fed diets for 15 weeks and tissue was isolated from animals sacrificed after a 6 hour fast. (n = 4, SC, LFD and HFD, n = 5 CAF). See Table S4 for full names of metabolites.

### Hierarchical Clustering and Heat Map Generation

Metabolites were selected that had complete data in at least 80% of experiments. Missing data was imputed using k-nearest neighbors with k = 8. SC was used as a reference, and each metabolite value was divided by the average metabolite value in SC diet. Ratios are presented as log_2_ (test diet/SC). For serum in Study 1, filtered metabolite data were analyzed by the one-class Significance Analysis of Microarrays (SAM) algorithm [37] to select the maximum set of metabolites whose expression levels were significantly different in test vs. SC given false discovery rate (FDR) of less than 5%. Data are presented as log2(fold change) relative to SC. Heat maps were generated using the R software package (http://www.r-project.org/).

**Figure 5:**
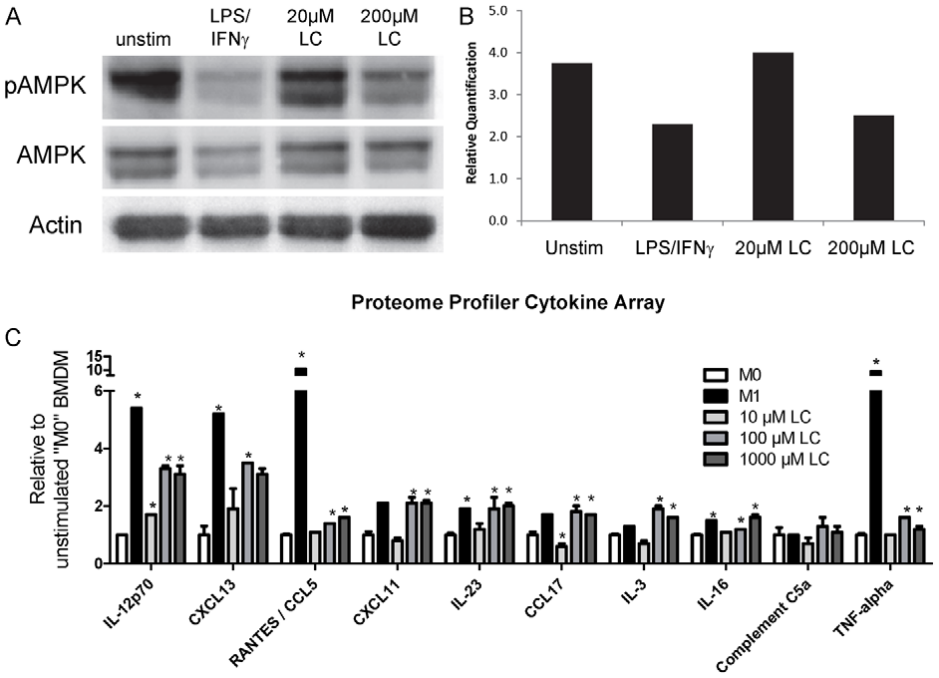
Lauroyl carnitine drives pro-inflammatory macrophage response. A) Primary bone marrow derived macrophages (BMDM) were treated with media alone for unpolarized macrophages “M0", 5 ng/mL LPS plus 10 ng/mL interferon gamma (IFNγ) to drive the pro-inflammatory “M1" phenotype [40], or 20 and 200 µM doses of lauroyl L-carnitine (“LC") for 24 hours. Western immunoblot using antibodies against phosphorylated AMP-activated protein kinase (AMPK), total AMPK, or actin. B) Bands are quantified using Image J and shown as pAMPK/AMPK normalized to actin. C) BMDM were treated for 24 hours with M1-polarizing cytokines (LPS/ IFNγ) or 10–1000 µM LC. Secreted cytokines were profiled from BMDM using Proteome Profiler Array Mouse Cytokine array (*p<0.05 relative to M0).

### Statistics, Principle Component Analysis, and Bioinformatics Analysis

First, serum, muscle, liver and white adipose metabolites were compared by analysis of variance (ANOVA) within each depot (serum or tissue). For each group, the list of p-values comparing test diets to control diet (one per metabolite) was then used to compute an FDR p-value. P-values adjusted for multiple comparisons less than 0.05 were considered statistically significant (all metabolite values and FDR p-values in Table S1, S2, and S3 for serum, muscle, and eWAT, respectively). All analyses were performed using SAS Version 9.2 (SAS Institute, Cary NC). Second, serum metabolites were examined by Principle Component Analysis (PCA) on each metabolite class. Pareto scaling was applied to the concentration data to normalize the effects of the large dynamic range of the metabolites [38], [39]. The cross validated standard error was calculated for each metabolite in the first PCA loadings component. If the magnitude of the error was less than the absolute value of the loadings, then the contribution of that metabolite was considered significant. The concentration of serum C2 acylcarnitine was not significantly different between the two diet groups, and therefore, it was excluded from the acylcarnitine model. Finally, Spearman pairwise correlation coefficients were calculated for each metabolite from study 1 with adjustments for multiple comparisons between serum metabolites and three parameters (blood glucose at time of sacrifice, weight gain, or homeostatic model assessment of insulin resistance (HOMA-IR) and significant correlations were presented using Benjamini-Hochberg FDR). For adipose tissue, Spearman pair-wise correlation coefficients were calculated with adjustments for multiple comparisons and significant correlations were presented using Benjamini-Hochberg FDR between metabolites and three parameters (blood glucose at the time of sacrifice, weight gain, or crown like structures). All Spearman statistical analyses were carried out using the R software package. Student's t-test was used to compare single measures where appropriate.

**Figure 6:**
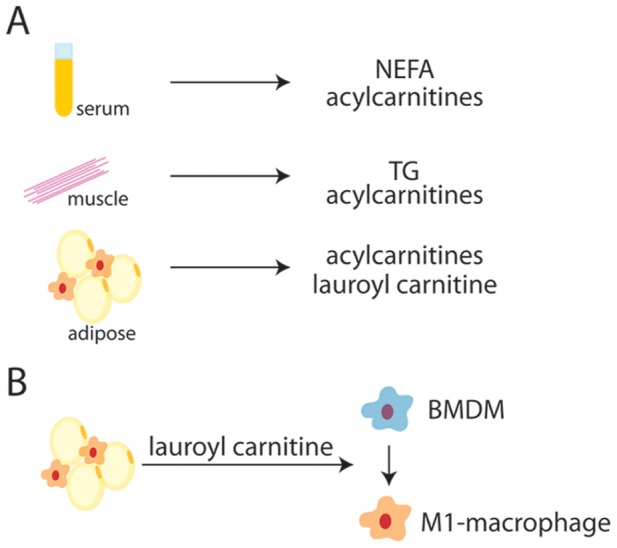
Cafeteria diet drives accumulation of oxidative intermediates and pro-inflammatory lipid mediators. Rats were fed SC or CAF diets for 10 weeks and at sacrifice serum and was isolated for metabolomic analysis including total and individual non-esterified fatty acids (NEFA), acylcarnitine and amino acid metabolite profiling of serum and muscle. To compare the effects of traditional defined lard-based diets with a CAF model on adipose metabolism, rats were fed SC, low fat and high fat lard-based defined diets, or CAF diet for 15 weeks and metabolites were analyzed. A. Metabolomic profiling has revealed that CAF diet-induced obesity drives accumulation of non-esterified fatty acids (NEFA), triglycerides (TG), and fatty acid β-oxidation intermediates in serum, muscle and for the first time white adipose tissue. CAF-mediated effects in adipose are more exaggerated than lard-based HFD effects. B. Lauroyl carnitine, an adipose-derived biomarker identified through metabolomic profiling was demonstrated to drive polarization of primary bone marrow derived macrophages (BMDM) towards the pro-inflammatory “M1" phenotype. Taken together, CAF diet proved to be a rapid and dramatic inducer of insulin resistance, components of Metabolic Syndrome, and metabolic biomarkers. In CAF-diet-induced obese adipose, lauroyl carnitine was identified as a potential mediator between metabolism of saturated fatty acids and the pro-inflammatory response.

### Lauroyl Carnitine Treatment of Macrophages

Bone marrow derived macrophages (BMDM) were isolated from 8 week old male C57Bl/6 mice, plated and cultivated on non-tissue culture treated 10 cm dishes for 6 days in RPMI-1640 supplemented with 30% L929 fibroblast (ATCC CCL-1)-conditioned media (containing M-CSF), 10% fetal bovine serum (FBS, Sigma-Aldrich, St. Louis, MO), 2 mM L-glutamine, and 100 units/mL penicillin G sodium, 100 μg/mL streptomycin sulfate (Life Technologies, Carlsbad, CA). BMDMs were trypsinized at day 6 and plated onto 10 cm tissue culture treated dishes. BMDM cells were treated the next day with fresh media for unpolarized macrophages (M0), 5 ng/mL lipopolysaccharide (LPS) plus 10 ng/mL interferon gamma (IFNγ) to drive the pro-inflammatory (M1) phenotype [40], or 20 and 200 µM doses of lauroyl (C12) L-carnitine (Avanti Polar Lipids, Inc., Alabaster, Alabama) for 24 hours. Protein lysates were isolated in 1% Triton-X 100, 0.05% sodium dodecal sulfate (SDS) and protease/phosphatase inhibitors (Sigma) and then quantified by BCA (Pierce) using a BioRad680 microplate reader and software.

### Western Immunoblot

Protein lysates were separated using SDS-polyacrylamide gel electrophoresis (PAGE) in a BioRad mini-PROTEAN Tetra-Cell unit, transferred to PVDF membranes using a BioRad Trans Blot Turbo transfer system, and blocked with 5% bovine serum albumin (BSA) in Tris-buffered saline with 0.2% Tween-20 (TBST) at room temperature for 1 hr. Primary antibodies were diluted according to the manufacturer guidelines in TBST with 5% BSA and applied to PVDF blots overnight at 4°C while rocking. Antibodies against phosphorylated AMP-activated protein kinase (AMPK), total AMPK (Cell Signaling, Danvers, MA), or actin (Millipore/Chemicon, Billerica, MA) were used according to the manufacturer's protocols. Horse radish peroxidase (HRP)-conjugated secondary goat-anti-rabbit antibodies and enhanced chemiluminescence (ECL) reagents (GE Healthcare Piscataway, NJ) were used to generate digital images acquired using the Versadoc multi-imaging system (BioRad). Bands were quantified using ImageJ software (BioRad) and reported as pAMPK/AMPK normalized to actin.

### Secreted Cytokine Profiling

BMDM were isolated as above, plated into 24 well plates and either treated with growth media for unstimulated macrophages (M0), 5 ng/mL LPS and 10 ng/mL IFNγ to polarize to the pro-inflammatory M1 phenotype, or 10–1000 μM lauroyl carnitine to mimic the obese adipose environment. After 24 hrs, conditioned media was isolated and assayed using Proteome Profiler Array Mouse Cytokine array panel A (R and D Systems, Inc. Minneapolis, MN) according to the manufacturer's protocol. Briefly, media was collected following treatments, centrifuged to remove cells and cellular debris at 14,000 g for 10 minutes, and the supernatant was frozen at−80°C until the assay was performed. 200 µL of media from pooled samples for each treatment group (n = 3) was then combined with the Detection Antibody Cocktail and allowed to mix 1hr, incubated overnight on membranes, and visualized with streptavidin-horse radish peroxidase (HRP) and ECL (GE Healthcare). Digital images were captured and quantified using the Versadoc 4000 (BioRad) and relative abundance with background subtracted was normalized to internal standards.

## Results

### Hierarchical clustering and principle component analysis of serum metabolites predict biomarkers of CAF-induced pathology

Serum metabolites can be predictive of insulin resistance and cardiovascular disease, including elevations in acylcarnitines and branched chain amino acids (BCAA) [28], [41]–[47]. Supervised cluster analysis was performed to elucidate patterns of serum metabolic changes in CAF vs. SC-fed animals. This cluster analysis implicates upregulation of many NEFAs and acylcarnitine species with downregulation of several amino acids, arachidonoyl carnitine (C20∶4), and short chain acylcarnitines in CAF-fed rats (FDR 3.88%, Fig. 1). Accumulation of acylcarnitines is indicative of inefficient beta-oxidation and mitochondrial dysfunction [27], [36]. All metabolite quantities and statistics are shown in Table S1.

To further identify relevant biomarkers associated with junk-food diet-induced obesity we used principal component analysis to identify clusters of metabolites that change coordinately in serum from rats exposed to the SC or CAF diets. Samples from each diet group are clustered together in each scores plot (principle component 1 (PC1), horizontal axis, Fig. S2A–C). Several fatty acids contribute to PC1, including the highly prevalent oleic acid (C18∶1) and linoleic acid (C18∶2). Interestingly, three of the eight NEFAs contributing to PC1 are pro-inflammatory saturated fatty acids elevated in the CAF diet-fed rodents: stearate (C18∶0), palmitate (C16∶0) and myristate (C14∶0) (Fig. 1 and Fig. S2A). Several acylcarnitine intermediates were identified that contributed to PC1 separation of SC vs. CAF including medium or short chain acylcarnitines such as dodecenoyl carnitine (C12∶1), tiglyl carnitine (C5∶1), tetradecenoyl carnitine (C14∶1), lauroyl carnitine (C12) and propionyl carnitine (C3) (Fig. 1 and Fig. S2B). All values and FDR p-values adjusted for multiple comparisons are presented in Table S1. Thus, PC1 is defined by widespread changes in a number of metabolic pathways, but with particularly strong changes in fatty acid metabolism.

### Dramatic weight gain in obese CAF-fed rats significantly elevates serum NEFA levels that correlate with physiologic measures of Metabolic Syndrome

Supervised cluster and PCA analysis of serum metabolites indicated that several serum NEFAs drove separation between SC and CAF-fed rats (Fig. 1 and Fig S1). Previous work by our group demonstrated elevated total NEFA levels in CAF-fed rats compared to HFD, LFD and SC-fed rats [12]. Here, we profiled individual NEFAs. Seven out of eight measured NEFA are significantly elevated in CAF-fed compared to SC-fed rats, including three saturated fatty acids (Fig. 2A). Spearman pairwise correlation coefficients were calculated to identify which metabolites predict the severity of Metabolic Syndrome phenotypes. Seventy-one metabolites were profiled from the serum metabolome including the sum NEFA, individual NEFAs, and multiple acylcarnitines and amino acids (Table S1). Metabolites were correlated with physiologic parameters including weight gain, blood glucose at time of sacrifice, and homeostatic model assessment of insulin resistance (HOMA-IR) as reported in Sampey et al. [12]. After adjustments for multiple comparisons between metabolites and physiologic measures, the elevated molar sum of serum NEFA in CAF-fed obese rats correlated weakly with HOMA-IR (r = 0.53, p = 0.029, FDR = 0.2), and most strongly with blood glucose (r = 0.71, p = 0.0013, FDR = 0.05) and weight gain (r = 0.73, p = 0.0012, FDR = 0.05) (Figures S2A–C, respectively). Among individual serum NEFAs, myristic acid (C14∶0) was most significantly correlated with weight gain (r = 0.81, p = 0.0001, FDR = 0.05), glucose (r = 0.66, p = 0.0038, FDR = 0.1), and HOMA-IR (r = 0.53, p = 0.027, FDR = 0.2) (Fig. 2B–D). Greater weight gain, glucose levels, and HOMA-IR are indicators of insulin resistance and Metabolic Syndrome [48]. Taken together our data suggest, at least in rat models, thresholds may be set including serum levels above 5 µM for myristic acid (C14∶0) and 225 µM for NEFAs above which elevated risk of components of Metabolic Syndrome is present. Interestingly, serum palmitoleic acid (C16∶1), a lipokine associated with improved insulin sensitivity and metabolic status in rodents and patients [49]–[51], was present at high levels in CAF-fed rats, and was positively correlated with poor metabolic status including weight gain (r = 0.60, p = 0.01, FDR = 0.1), elevated blood glucose (r = 0.61, p = 0.01, FDR = 0.1), and high NEFA levels (r = 0.84, p = 0.00003, FDR = 0.05) (correlation images not shown).

### Metabolic markers of saturated fatty acids and other biomarkers correlate with poor metabolic status

Just as the serum NEFA myristate (C14∶0) correlated with markers of Metabolic Syndrome (Fig. 2B–D), elevated levels of serum AC derived from myristic acid (myristoyl carnitine, C14–AC) also significantly correlated with markers of poor metabolic status including blood glucose (r = 0.63, p = 0.007, FDR = 0.1), NEFA (r = 0.56, p = 0.02, FDR = 0.1), and weight gain (r = 0.56, p = 0.02, FDR = 0.2). Another serum saturated fatty acid, lauric acid was not independently measured in this study, but the lauric acid-derived serum acylcarnitine (lauroyl carnitine, C12-AC) was elevated in CAF-fed rat serum and correlated with blood glucose (r = 0.56, p = 0.02, FDR = 0.2) and weight gain (r = 0.65, p = 0.005, FDR = 0.1) (values reported in Table S1, correlation images not shown).

Alterations in acylcarnitines derived from branched chain amino acid (BCAA) catabolism are reflected by changes in small chain acylcarnitines C3, C4, C5 and related metabolites [28]. Cluster analysis of serum in Figure 1 suggested that C3, C4 and C5 metabolites are relevant in CAF versus SC comparisons and are downregulated by CAF diet exposure. C5 acylcarnitines are comprised of α-methylbutyrylcarnitine and isovalerylcarnitine species which equilibrate with intermediates in BCAA isoleucine and leucine catabolism, respectively, while C3 acylcarnitine represents propionyl CoA, an intermediate of isoleucine and valine catabolism. Interestingly, in our CAF diet model, PCA identified C3-AC and C5-DC as serum biomarkers delineating the SC and CAF groups (Fig. S1B). Serum propionyl carnitine (C3-AC) was consistently decreased by CAF feeding (Fig. 1 and Tables S1, S2, S3, although this difference only reached statistical significance by ANOVA accounting for multiple comparisons in muscle). Interestingly, plasma C3 and C5 acylcarnitines are elevated in plasma of obese and insulin resistant humans in concert with elevations in BCAA, and feeding a HFD actually causes these intermediates to decrease in plasma [28], consistent with the current findings. However, our data also shows that levels of C3 acylcarnitines are significantly increased in skeletal muscle of CAF-fed rats, consistent with the concept that BCAA can contribute to overload of mitochondrial metabolism [28], [52].

### Diet-induced mitochondrial dysfunction was evident in muscle of CAF-fed rats

Muscle lipid accumulation has been associated with mitochondrial dysfunction in mice made obese by a traditional lard-based HFD [36]. To examine if CAF-diet also induced mitochondrial dysfunction, acylcarnitine profiles were evaluated in muscle compared to SC-diet. We have previously published that rats fed CAF diet gained an average of 100 g more than SC after just 10 weeks on diet, with substantial lipid accumulation in liver, white and brown adipose tissue [12]. CAF-fed white and brown fat pads were 3-fold heavier than SC-fed rats and contained elevated macrophage infiltration [12]. In addition, livers of CAF-fed rats displayed severe pan-lobular microsteatosis and large inflammatory loci [12]. Here we also report that after 10 weeks on diet, muscle triglyceride content was elevated by two-fold compared to SC-fed rats (Fig. 3A, p = 0.017). Furthermore, Figure 3B demonstrates accumulation of a subset of muscle acylcarnitines comprised of short, medium and long chain species in CAF-fed rodents compared to SC-fed controls indicative of mitochondrial dysfunction, as was also found in serum (Fig. 1 and Table S1). All muscle metabolite quantities and statistics are shown in Table S2. Liver metabolites did not demonstrate significant alterations in metabolic profiles of AA, OA, or AC by ANOVA (data not shown). Note the aforementioned increase in muscle C3 acylcarnitines in CAF-fed animals.

### Adipose tissue displayed diet-induced mitochondrial dysfunction

We hypothesized that metabolism in adipose tissue might be defective as it is the major tissue associated with storage and release of lipids as well as chronic obesity-induced inflammation. Therefore, we next completed a 15 week diet study on rats fed four different diets. Our previous reports demonstrate that epididymal white adipose tissue (eWAT) from CAF-fed animals are inflamed and display elevations in inflammatory markers such as macrophage aggregates (crown-like structures) and elevated pro-inflammatory cytokine TNFα expression in the macrophage-enriched stromal-vascular fraction [53]. In addition to CAF and SC diets as above, rodents were fed two additional diets most frequently used in diet-induced obesity studies: 10% kcal-derived from fat (low fat diet, LFD); and 45% kcal-derived from lard-based fat (high fat diet, HFD) so that CAF diet and its control can be compared directly to HFD and LFD. The CAF-fed rats gained nearly 250 grams more weight than SC-fed rats over 15 weeks [12]. LFD and HFD rats displayed metabolic parameters and gained weight in an intermediate range compared to SC and CAF diet-exposed animals (75 g and 100 g greater than SC, respectively) [12]. After 15 weeks on diet, eWAT was isolated and metabolomic profiling of amino acids, organic acids, acylcarnitines, and free carnitine was conducted on fat (Table S3). CAF diet induced dramatic mitochondrial dysfunction in eWAT as evidenced by increased levels of multiple medium and long chain acylcarnitines when compared to SC-fed control animals with fewer alterations evident in HFD-fed eWAT acylcarnitines versus LFD (Fig. 4A and Table S3). ANOVA analysis of the four diets revealed lauroyl carnitine (C12-AC) as the most significantly regulated metabolite with levels in CAF-fed rodent adipose over two-fold greater than in HFD, LFD or SC-fed tissue (false discovery rate (FDR) p-value adjusted for multiple comparisons p = 0.00069). Compared to LFD, HFD feeding significantly increased acylcarnitines C16∶0, C18∶0 and C18∶1, while CAF-diet exposure increased C10, C12, C18∶1 and C18 compared to SC (Fig. 4A and Table S3).

### Adipose lauroyl carnitine correlates with physiologic and histologic measures of Metabolic Syndrome and inflammation

Spearman pairwise correlation coefficients were calculated with adjustments for multiple comparisons between metabolites and physiologic measures of blood glucose at time of sacrifice, weight gain, or NEFA. Adipose lauric acid acylcarnitine (lauroyl carnitine, C12-AC) correlated significantly with blood glucose (r = 0.76, p = 0.0004, FDR = 0.1). In our previous work, we examined histologic markers of adipose inflammation denoted by crown like structures (CLS), which have been shown to correlate with obesity, insulin resistance, and adipose inflammation. CLS per 10X field were 15.3-, 2.75-, 1.5-fold higher in CAF diet-fed rat fat pads compared to SC, LFD and HFD, respectively [12]. Spearman pairwise correlations revealed that lauroyl carnitine also positively correlated with CLS (r = 0.69, p = 0.002, FDR = 0.05, Fig. 4B). Interestingly, lauroyl carnitine did not correlate with inflammatory loci in livers (not shown and Sampey et al. [53]). Overall, in our model, levels above 5 pmol/mg tissue for lauroyl carnitine in epididymal white adipose correlated with risk factors for Metabolic Syndrome.

### Lauroyl carnitine-mediated inflammation of BMDM

The identification of the NEFA laurate and acylcarnitine metabolite lauroyl carnitine as biomarkers associated with obesity, insulin resistance, and inflammation in CAF-fed rodents led us to investigate if lauroyl carnitine plays a causal role in these associations. Macrophages are an integral component of adipose tissue with less inflammatory macrophages resident in lean tissue and pro-inflammatory macrophages infiltrating with increasing obesity [6], [7], [54]. Since lauroyl carnitine was specifically elevated in CAF-fed adipose tissue, we treated primary BMDM with increasing doses of lauroyl carnitine to provide mechanistic insight regarding macrophage inflammation in adipose tissue. Resident M2 macrophages are reliant upon fatty acid metabolism and AMPK activity [40], [55]. Sag et al. demonstrated that activation of AMPK (i.e. phosphorylation) drives anti-inflammatory polarization in macrophages [55]. Figure 5 demonstrates that 24 hours of LPS and IFNγ pro-inflammatory M1 polarization of BMDM caused a clear decrease in AMPK phosphorylation. Treatment with 200 µM lauroyl carnitine also caused a decrease in AMPK phosphorylation to levels similar to those in M1 BMDMs. Cytokine array analyses of conditioned media revealed that M1 polarization of BMDMs drove expression of the pro-inflammatory cytokines TNFα, RANTES (CCL5), IL-6, CXCL9, and CCL4 (not shown and Fig. 5C). Interestingly, lauroyl carnitine increased expression of several cytokines, in a dose-dependent fashion that approximated levels present with cytokine-stimulated M1 polarization of BMDM. While lauroyl carnitine did not dramatically regulate all cytokines associated with obesity and inflammation, it did upregulate the pro-inflammatory cytokines IL-3, IL-12, IL-16, IL-23, CXCL11, CXCL13, RANTES, and CCL17 (Fig. 5C) that primarily regulate chemotaxis, demonstrating a direct C12-AC-mediated activation of pro-inflammatory cytokines.

## Discussion

Recent work has demonstrated that metabolomic analysis can provide a view of mitochondrial function that reflects the physiologic or pathophysiologic state of the system [26], [27], [36], [56]. Acylcarnitines have traditionally been used to measure inborn errors of metabolism, but in recent years have been shown to be markers of insulin resistance [24]. Acylcarnitines in particular are valuable biomarkers because they represent byproducts of fat, glucose, and amino acid mitochondrial oxidation. The work presented here contributes to increasing evidence that elevated acylcarnitines may act as biomarkers of insulin resistance, cardiovascular disease (CVD), defects in fat metabolism, as well as inflammation in Metabolic Syndrome [27], [28], [36], [43]. We further provide evidence and mechanistic insight of how one biomarker, lauroyl carnitine, may mediate inflammation associated with obesity in adipose.

We observed that CAF-fed animals had higher levels of pro-inflammatory saturated fatty acids such as C12 (laurate) and C14 (myristate), as well as the acylcarnitine derivatives lauroyl carnitine and myristoyl carnitine. Obesity-associated increases in circulating fatty acids cause functional impairments in several cell types including hepatocytes, skeletal muscle, cardiomyocytes, β-cells, endothelial cells and adipocytes [57]. Emerging evidence also implicates mitochondrial dysfunction as a potentially unifying mechanism underlying these observations. Koves et al. have shown that obesity and/or overnutrition impose a persistent lipid burden on muscle mitochondria, resulting in a mismatch between β-oxidation and tricarboxylic acid cycle activity [58]. This disconnect results in high rates of incomplete fatty acid β-oxidation and increases in acylcarnitines, which correlated with impaired muscle insulin sensitivity and glucose metabolism [26]. Acylcarnitine accumulation, or mitochondrial dysfunction, can be improved with exercise or in rodent models of improved glucose tolerance [26], [58]. In line with our observations, others have shown saturated fatty acids such as myristate correlate with obesity, cholesterol, and lower levels of adiponectin in human populations [59]–[62]. In contrast, some have shown fatty acids such as palmitoleic acid to attenuate insulin resistance in mouse models and correlate with some beneficial lipid measures in human studies [49]–[51]; interestingly in our rat model palmitoleic acid was positively correlated with markers of Metabolic Syndrome and insulin resistance, which suggests that this may be a species-specific finding.

It is well-accepted that obese adipose tissue is in a state of persistent low level inflammation and that increasing obesity drives further inflammation and subsequent insulin resistance [63]. Although strong links between dyslipidemia, obesity, and inflammation are established, little is known about mediators of adipose inflammation [3], [4]. We demonstrated that lauroyl carnitine, a biomarker identified through metabolomics, is one such mediator. Lauroyl carnitine inhibited AMPK activation, a pathway known to promote an anti-inflammatory M2 macrophage phenotype associated with insulin sensitivity [55]. In fact, the inhibition was similar to that observed with M1 polarization. Interestingly, Garvey et al. recently showed in the RAW264.7 macrophage cell line that lauroyl carnitine can activate NFΚB signaling [64]. Using a cytokine profiling assay, we further report increased pro-inflammatory and chemotactic cytokine secretion following lauroyl carnitine exposure in primary BMDMs that reflects the M1 proinflammatory phenotype. Thus, our findings suggest that lauroyl carnitine may act as a pro-inflammatory lipokine through AMPK pathway de-activation. Future studies will need to be conducted to determine *in vivo* relevance of the role of lauroyl carnitine on adipose microenvironment inflammation.

While the effects of lauroyl carnitine appear to be profound, several mechanistic details remain uncertain. Signaling through pattern recognition receptors including TLRs and GPCRs have been implicated in saturated fatty acid-mediated inflammation, especially lauric acid TLR-mediated activation of NFΚB through the MyD88 pathway [23], [65]–[77]; it is unclear if lauroyl carnitine can also activate these receptors. It is likely that CAF-diet induces a feed-forward loop which includes increased intake of saturated fatty acids, as well as elevated caloric intake in general, which together act to drive obesity, release the pro-inflammatory metabolite lauroyl carnitine, and promote inflammation concurrently. Hence the elevated acylcarnitines present in CAF-fed rats, both systemically and in tissues, are not only markers of the potential inflammatory state, but may act as mediators to specifically promote inflammation.

In summary, this study included a comprehensive analysis of physiologic, metabolic, and histologic measures between a commonly used dietary model for diet-induced obesity, lard-based HFD, and its LFD control, compared to an alternative human junk food-based dietary model, the CAF diet. Findings presented herein and in our previous work demonstrate that the CAF diet results in a more dramatic phenotype of obesity and related metabolic abnormalities compared to traditional HFD [12]. Compared to traditional lard-based diets commonly used in rodent diet-induced obesity studies, which are high in saturated fatty acids but also polyunsaturated fatty acids, CAF diet provides many components associated with Metabolic Syndrome, including fat (saturated and trans-fats), sodium, and cholesterol, plus is low in protective nutrients such as fiber and micronutrients. While the effects of CAF diet are dramatic, no causal associations can be inferred for a specific component in our studies. Figure 6 summarizes our findings: metabolomic analysis identified a CAF biomarker signature consistent with rapid-onset Metabolic Syndrome and elevated mitochondrial dysregulation that corresponds to weight gain, HOMA-IR measures of insulin sensitivity, hyperglycemia, pro-inflammatory macrophage infiltration of adipose and crown-like structure formation. Further, we demonstrate that one metabolite that was significantly elevated in CAF-adipose tissue, lauroyl carnitine, can drive the pro-inflammatory activation of macrophages. The CAF diet, while not traditionally utilized, may be superior to HFD to model modern human obesity trends including exposure to energy-dense, nutrient-poor diets, early and rapid obesity development, and elevated markers of Metabolic Syndrome and inflammation.

## Supporting Information

Figure S1 **Principle component (PC) analysis of the serum metabolome.** Principle component analysis for each metabolite class was carried out, NEFA, acylcarnitine and amino acids (A–C, respectively). The scores plots reveal a distinct metabolic perturbation in each metabolite class between the standard chow- (SC, white square) and the Cafeteria- (CAF, black circle) fed rat samples primarily along the first component 1. The percent variation explained by the two principle components for each model is shown on the axes in parentheses. See Table S4 for full names of acylcarnitines and amino acids. (n = 8 SC, 9 CAF).
(TIF)

Figure S2 **NEFAs are elevated in CAF-fed rats and correlate with markers of Metabolic Syndrome: HOMA-IR, blood glucose, and weight gain.** The molar sum of NEFAs significantly correlates with HOMA-IR (A), blood glucose at sacrifice (B), and weight gain (C). Aged-matched male rats were fed SC or CAF diets for 10 weeks and serum was isolated in 6 hour fasted rats. (n = 8 SC, 9 CAF).
(TIFF)

Table S1 **Serum metabolites.** Numbers are mean ± SEM. N = 8 for SC and 9 for CAF. P-values are calculated by ANOVA, false discovery rate (FDR) p-value adjusted for multiple comparisons (empty wells are non-significant).
(TIFF)

Table S2 **Muscle tissue metabolites.** Numbers are mean ± SEM. N = 12 for SC and 9 for CAF. P-values are calculated by ANOVA, false discovery rate (FDR) p-value adjusted for multiple comparisons (empty wells are non-significant).
(TIFF)

Table S3 **Epididymal white adipose tissue metabolites.** Numbers are mean ± SEM. N = 4 for all groups, except n = 5 in CAF. P-values are calculated by Student's t-test (empty wells are non-significant).
(TIFF)

Table S4 **Detailed biochemical names and metabolite symbols.**
(TIFF)
