# Supplementary figures and images for: Metabolomic Profiling Reveals Mitochondrial-Derived Lipid Biomarkers That Drive Obesity-Associated Inflammation
Source: PLoS One. 2012 Jun 12;7(6):e38812. doi: 10.1371/journal.pone.0038812 (PMC3373493; doi:10.1371/journal.pone.0038812)

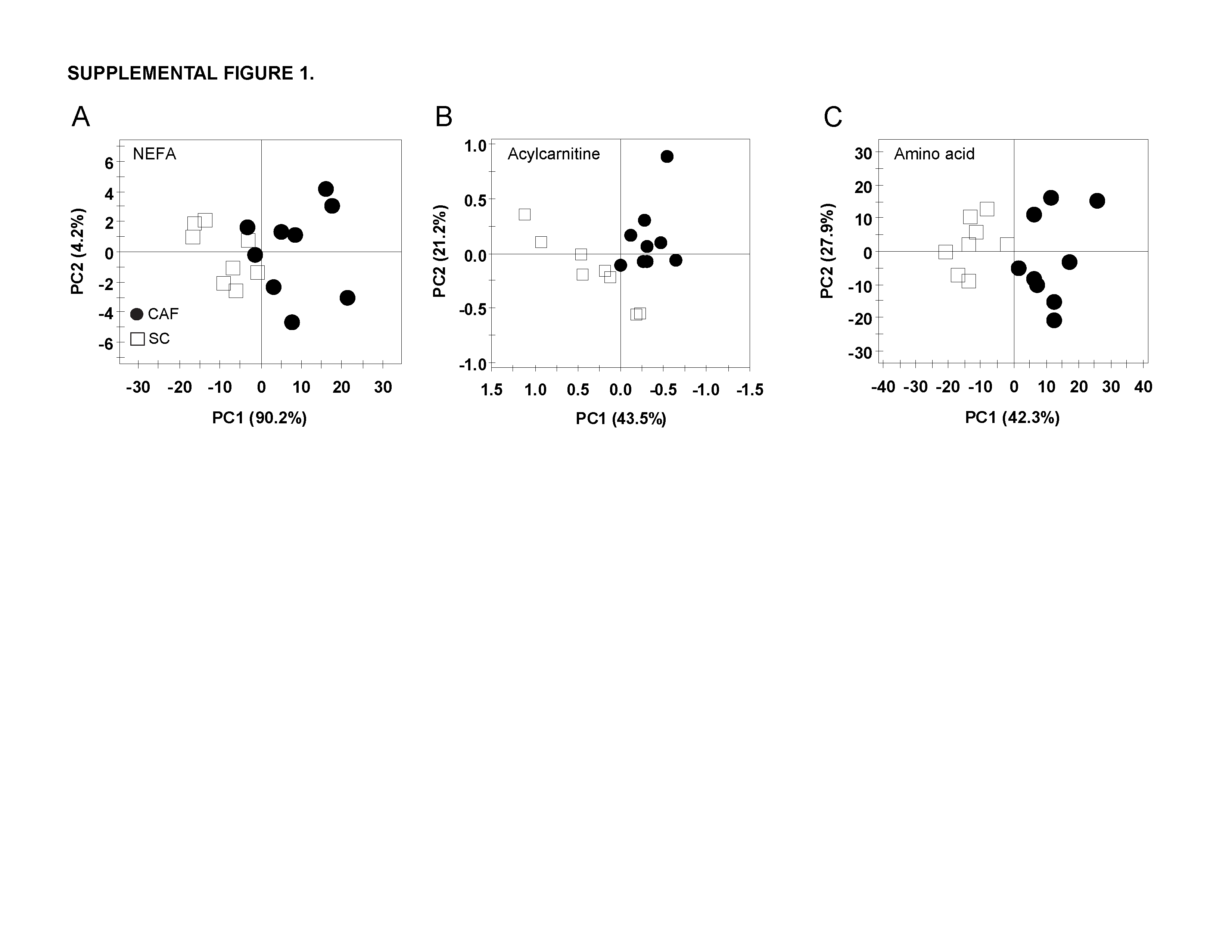

Supplement: Figure S1 — Principle component (PC) analysis of the serum metabolome. Principle component analysis for each metabolite class was carried out, NEFA, acylcarnitine and amino acids (A–C, respectively). The scores plots reveal a distinct metabolic perturbation in each metabolite class between the standard chow- (SC, white square) and the Cafeteria- (CAF, black circle) fed rat samples primarily along the first component 1. The percent variation explained by the two principle components for each model is shown on the axes in parentheses. See Table S4 for full names of acylcarnitines and amino acids. (n = 8 SC, 9 CAF). (TIF) [file pone.0038812.s001.tif]

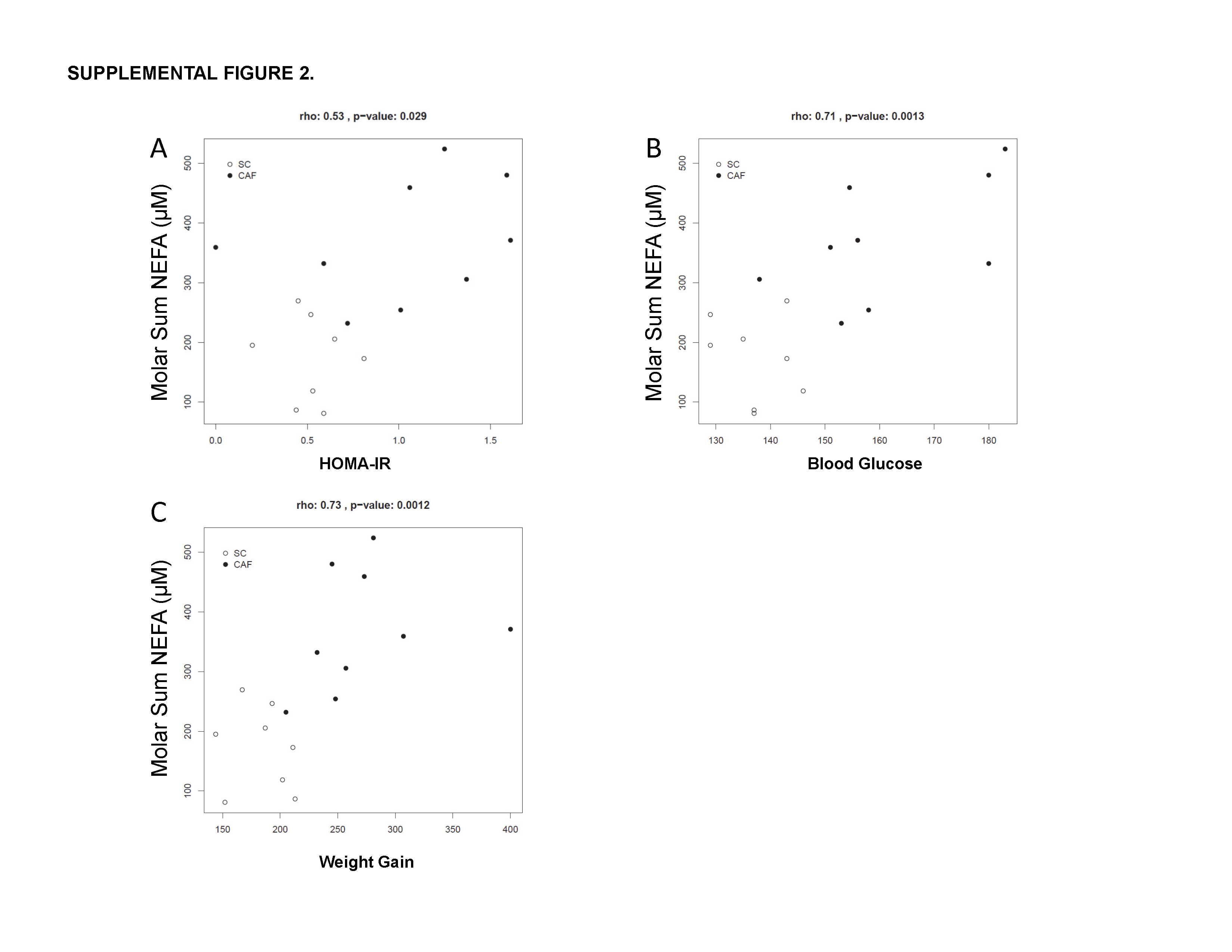

Supplement: Figure S2 — NEFAs are elevated in CAF-fed rats and correlate with markers of Metabolic Syndrome: HOMA-IR, blood glucose, and weight gain. The molar sum of NEFAs significantly correlates with HOMA-IR (A), blood glucose at sacrifice (B), and weight gain (C). Aged-matched male rats were fed SC or CAF diets for 10 weeks and serum was isolated in 6 hour fasted rats. (n = 8 SC, 9 CAF). (TIFF) [file pone.0038812.s002.tif]

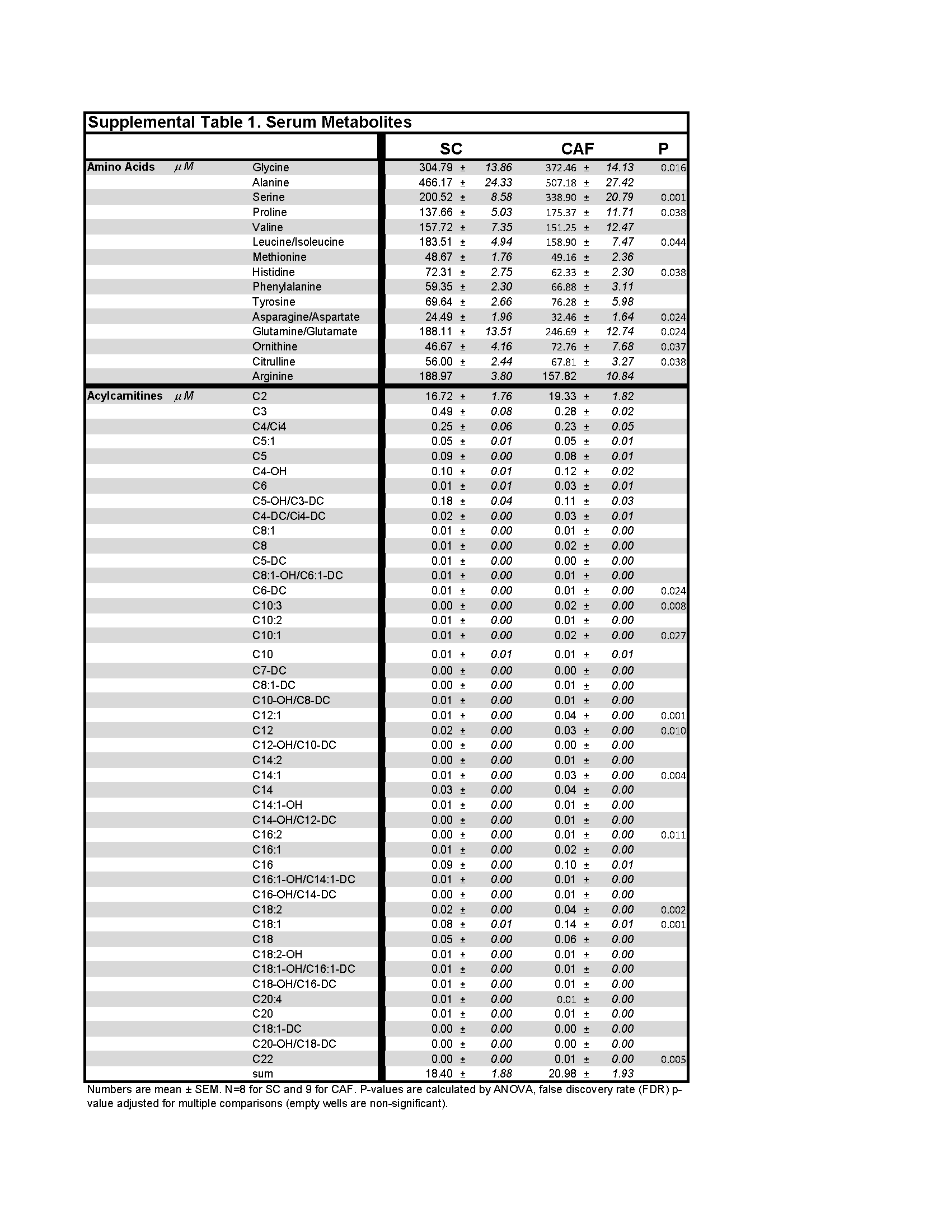

Supplement: Table S1 — Serum metabolites. Numbers are mean ± SEM. N = 8 for SC and 9 for CAF. P-values are calculated by ANOVA, false discovery rate (FDR) p-value adjusted for multiple comparisons (empty wells are non-significant). (TIFF) [file pone.0038812.s003.tif]

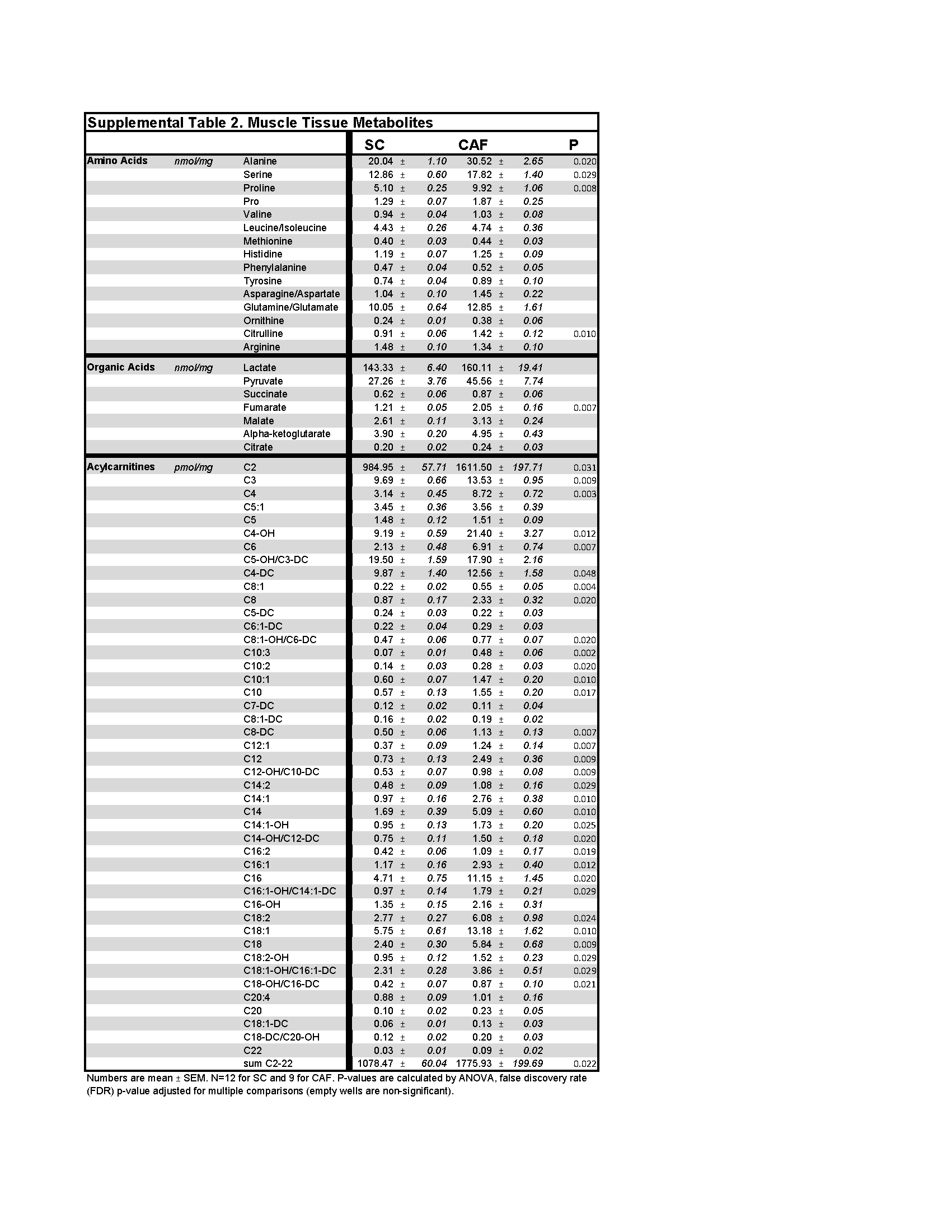

Supplement: Table S2 — Muscle tissue metabolites. Numbers are mean ± SEM. N = 12 for SC and 9 for CAF. P-values are calculated by ANOVA, false discovery rate (FDR) p-value adjusted for multiple comparisons (empty wells are non-significant). (TIFF) [file pone.0038812.s004.tif]

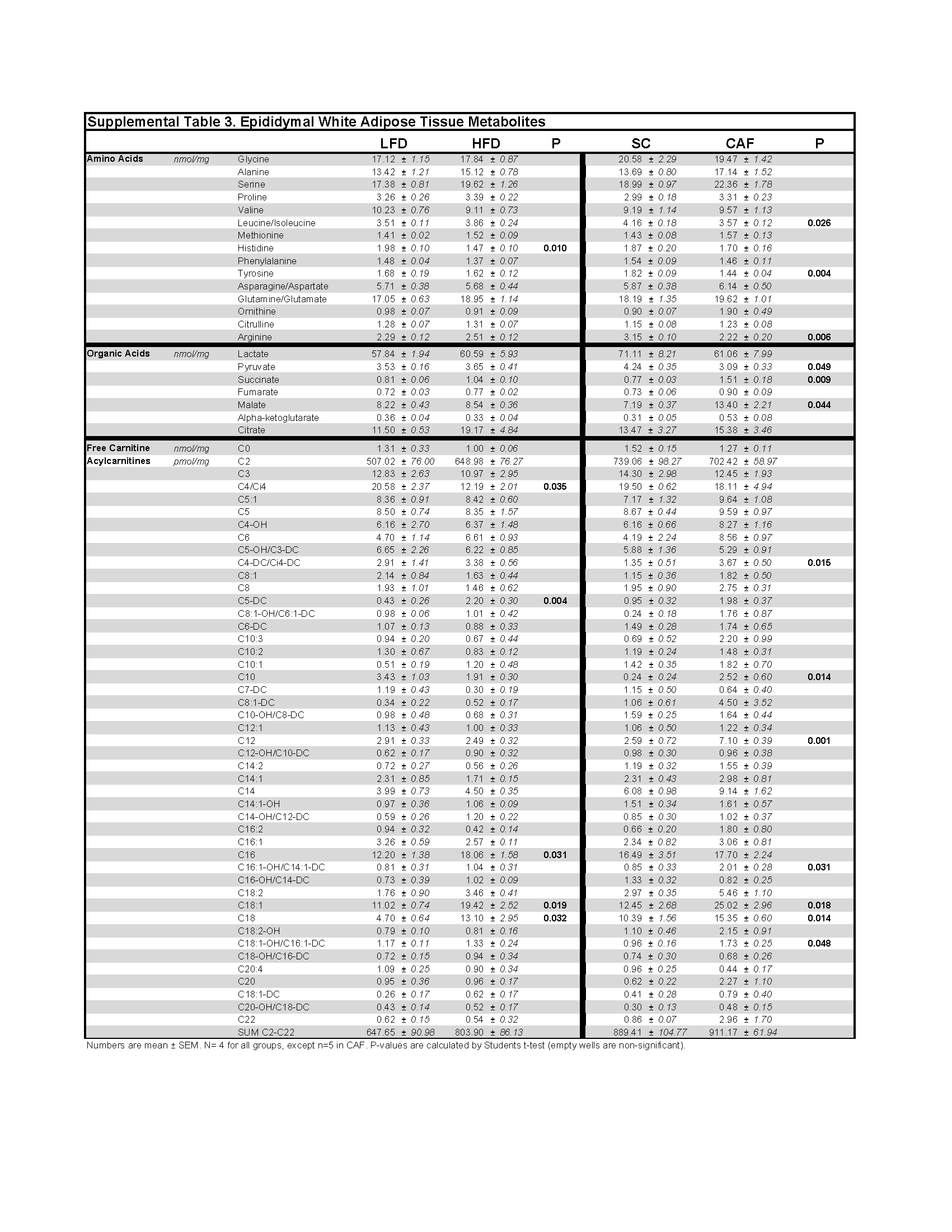

Supplement: Table S3 — Epididymal white adipose tissue metabolites. Numbers are mean ± SEM. N = 4 for all groups, except n = 5 in CAF. P-values are calculated by Student's t-test (empty wells are non-significant). (TIFF) [file pone.0038812.s005.tif]
